# Early Marriages

**Published:** 1862-11

**Authors:** 


### EARLY MARRIAGES.

Those earnest and practical moralists who, having seen a source of national enfeeblement and degradation in the gradually widening proportions of the social evil, have essayed to check it, point justly to early marriages as one remedy. In a valuable article on the Midnight Mission, *The Times* returns to this important subject. Public opinion needs to be turned in this direction; and if some share of the attention be bestowed on it by earlier legislators and philosophers were now once more aroused, a step would be taken towards healing this cancer of our civilization. Aristotle, in the eighth book of his "Politics," elaborately discusses the question of age, and fixes the term in which statesmen should enjoin and beyond which they should forbid marriage. Eighteen he names as the fit age of the female, and twenty-seven for the male sex. In Rome, vicious and debased as it was, the *lex Poppæa* prohibited from marriage men of sixty and women of fifty. On the other hand, precocious unions were discouraged among the Lacedæmonians and other nations of antiquity. Physiologically, the age for marriage of the male may be fixed at from twenty-two to twenty-five, and from seventeen to twenty-one of the female. "Children born too late are orphans too early," says the Spanish proverb; but it must be added that they do not survive in due proportions to verify that unpleasant prediction. Nor is anything more miscalculated than the sort of *equilibration* which is sometimes essayed by the union of age and exhaustion on the one side with youth and vigor on the other. The results are exactly opposite to those which we may suppose to be sought, and a puny and vitiated offspring proclaim the ignorant infraction of one of Nature's first laws. There is no compensation to be obtained in this manner. The early years of fashionable profligacy which now form the usual novitiate of the prosperous classes threaten an undoubted degeneration of our race. Of late years an open toleration has been extended to these habits, of which the bad effects are already widely apparent to medical observers. The evil spreads and becomes more pressing.

"Æstas perantum pejor avis tulit
Nos nequiores mox daturos
Progeniem vitiosiorem."

This toleration may at least be revoked; something may be done to make the paths of vice less flowery, and to facilitate those early and happy unions which offer so many moral and physical advantages.—*London Lancet.*

[The subjoined came to hand too late for insertion in its proper place.—Ed.]

Madison, Ind., Sept. 4th, 1862.

*Messrs. Editors :*—Dear Sirs : Having noticed the different operations of Dr. R. R. Browne, reported in the August number of your valuable journal, I find that he is at a loss to know what to use for the removal of maggots from the wounds of our brave soldiers. I would suggest the use of Turpentine. I have used it repeatedly in my practice without ever failing. It also cleanses the parts of all dead matter, and seems to produce a healthy action and heals more readily than anything I have ever tried. I have also used Honey. It is not as certain as the Turpentine. W. J. Conner, M. D.

